# Pulmonary manifestations in VEXAS (vacuoles, E1 enzyme, X-linked, autoinflammatory, somatic) syndrome: a systematic review

**DOI:** 10.1007/s00296-022-05266-2

**Published:** 2023-01-08

**Authors:** Koushan Kouranloo, Athea Ashley, Sizheng Steven Zhao, Mrinalini Dey

**Affiliations:** 1 School of Medicine, University of Liverpool, Ashton Street, Liverpool, L69 3GE UK; 2 Liverpool University NHS Foundation Trust, Prescot Street, Liverpool, L7 8XP UK; 3 Bradford Royal Infirmary, Duckworth Lane, Bradford, BD9 6RJ Yorkshire UK; 4 Versus Arthritis Centre for Epidemiology, Centre for Musculoskeletal Research, The University of Manchester, Manchester, M13 9PL UK; 5 Department of Rheumatology, Queen Elizabeth Hospital, Stadium Rd, London, SE18 4QH UK; 6 Institute of Life Course and Medical Sciences, University of Liverpool, Brownlow Hill, Liverpool, L69 3BX UK

**Keywords:** VEXAS, Autoinflammatory syndrome, Autoimmune disease, Respiratory, Pulmonary

## Abstract

**Background:**

VEXAS (vacuoles, E1 enzyme, X-linked, auto-inflammatory, somatic) syndrome is a newly described auto-inflammatory disease. Many cases feature pulmonary infiltrates or respiratory failure. This systematic review aimed to summarize respiratory manifestations in VEXAS syndrome described to date.

**Methods:**

Databases were searched for articles discussing VEXAS syndrome until May 2022. The research question was: What are the pulmonary manifestations in patients with VEXAS syndrome? The search was restricted to English language and those discussing clinical presentation of disease. Information on basic demographics, type and prevalence of pulmonary manifestations, co-existing disease associations and author conclusions on pulmonary involvement were extracted. The protocol was registered on the PROSPERO register of systematic reviews.

**Results:**

Initially, 219 articles were retrieved with 36 ultimately included (all case reports or series). A total of 269 patients with VEXAS were included, 98.6% male, mean age 66.8 years at disease onset. The most frequently described pulmonary manifestation was infiltrates (43.1%; *n* = 116), followed by pleural effusion (7.4%; *n* = 20) and idiopathic interstitial pneumonia (3.3%; *n* = 9). Other pulmonary manifestations were: nonspecific interstitial pneumonia (*n* = 1), bronchiolitis obliterans (*n* = 3), pulmonary vasculitis (*n* = 6), bronchiectasis (*n* = 1), alveolar haemorrhage (*n* = 1), pulmonary embolism (*n* = 4), bronchial stenosis (*n* = 1), and alveolitis (*n* = 1). Several patients had one or more co-existing autoimmune/inflammatory condition. It was not reported which patients had particular pulmonary manifestations.

**Conclusion:**

This is the first systematic review undertaken in VEXAS patients. Our results demonstrate that pulmonary involvement is common in this patient group. It is unclear if respiratory manifestations are part of the primary disease or a co-existing condition. Larger epidemiological analyses will aid further characterisation of pulmonary involvement and disease management.

**Supplementary Information:**

The online version contains supplementary material available at 10.1007/s00296-022-05266-2.

## Introduction

Vacuoles, E1 enzyme, X-linked, auto-inflammatory, somatic (VEXAS) syndrome is a recently described autoinflammatory condition [1]. First reported in 2020, it is due to somatic mutations in the UBA1 gene of myeloid cells and mainly affects older men. The disease is characterized by inflammatory and haematologic symptoms, arising from myeloid-driven autoinflammation and progressive bone marrow failure [2].

The systemic inflammation in patients with VEXAS syndrome can affect multiple organs, most commonly the skin, blood vessels, cartilage and lungs. This often leads to co-existing or misdiagnoses of other systemic rheumatic and autoinflammatory disorders such as Sweet syndrome, relapsing polychondritis and medium-large vessel vasculitides.

Features of patients with VEXAS syndrome continue to be under investigation and comprise a spectrum of phenomena arising from systemic inflammation. One of the most pertinent and consistent feature is pulmonary involvement, as for many autoinflammatory conditions [3–5]. Since VEXAS was first described, case reports and small cohort studies have reported various respiratory manifestations in a large proportion of this patient group including pulmonary embolism, parenchymal disease and pulmonary vasculitis [6–8]. Lung involvement is often severe, leading to significant morbidity and mortality [9].

To date, no systematic review has been undertaken of reported cases of VEXAS, or pulmonary manifestations of this disease. This systematic review aimed to summarize the respiratory manifestations of VEXAS syndrome, described in the literature.

## Methods

This systematic review was conducted in accordance with the Cochrane Handbook and reported as per the Preferred Reporting Items for Systematic Reviews and Meta-Analyses guidelines [10, 11]. The protocol was developed by KK, SZ and MD, and registered in the PROSPERO database of systematic reviews (CRD42022355163). The research question was framed and structured using the ‘Patients, Intervention, Comparator or Control and Outcome’ (PICO) format: What are the pulmonary manifestations described in VEXAS syndrome?

The population was defined as adult patients with a clinician-confirmed diagnosis of VEXAS. The main outcome was pulmonary manifestations including (but not limited to): pulmonary infiltrates; idiopathic interstitial pneumonia; non-specific interstitial pneumonia (NSIP); bronchiolitis obliterans; pulmonary vasculitis; bronchiectasis; pleural effusion; alveolar haemorrhage; pulmonary embolism (and related MeSH terms). “Intervention” and “comparator” were not applicable to the search.

### Search strategy, databases searched and study selection

The search strategy (available in the supplementary material) was developed by two authors (KK and MD) with the help of a librarian expert in undertaking systematic reviews and clinical research (HE). The bibliographic databases Medline, Scopus, The Cochrane Library (the Cochrane Database of Systematic Reviews, the Cochrane Central Register of Controlled Trials (CENTRAL), and the Cochrane Methodology Register), and PsycINFO were searched via the Ovid platform on 13 May 2022. The search conducted had no time restriction and was limited to English-language only. All study types, excluding opinion articles, editorials and literature reviews.

Due to the relative recency of VEXAS syndrome being described in clinical practice, the search strategy was kept deliberately broad to ensure all relevant articles were captured. The ultimate focus of the review was pulmonary manifestations described in VEXAS syndrome.

Titles and abstracts were screened by AA and MD, to assess eligibility. The full articles which met the inclusion criteria were then examined in detail by AA. For validation purposes, 10% of the articles were screened at the abstract and full paper stage by a second author, KK, with input from a third reviewer (MD) where required.

### Assessment of risk of bias, data extraction and synthesis

Risk of bias in each included study was assessed using the appropriate tool for each study. Risk of bias was not possible for case reports and studies. Data extraction from the included articles was undertaken by AA, with 10% of articles also reviewed and the information extracted by KK for validation. No papers or additional data or online supplemental material were required from authors.

For each selected article, in addition to basic information and patient demographics, the following were extracted: number of reported cases with lung involvement; percentage of lung involvement and their subtypes (if described); ethnicity of sample (if available); comorbidities; and summary of authors’ conclusions.

## Results

**Fig. 1:**
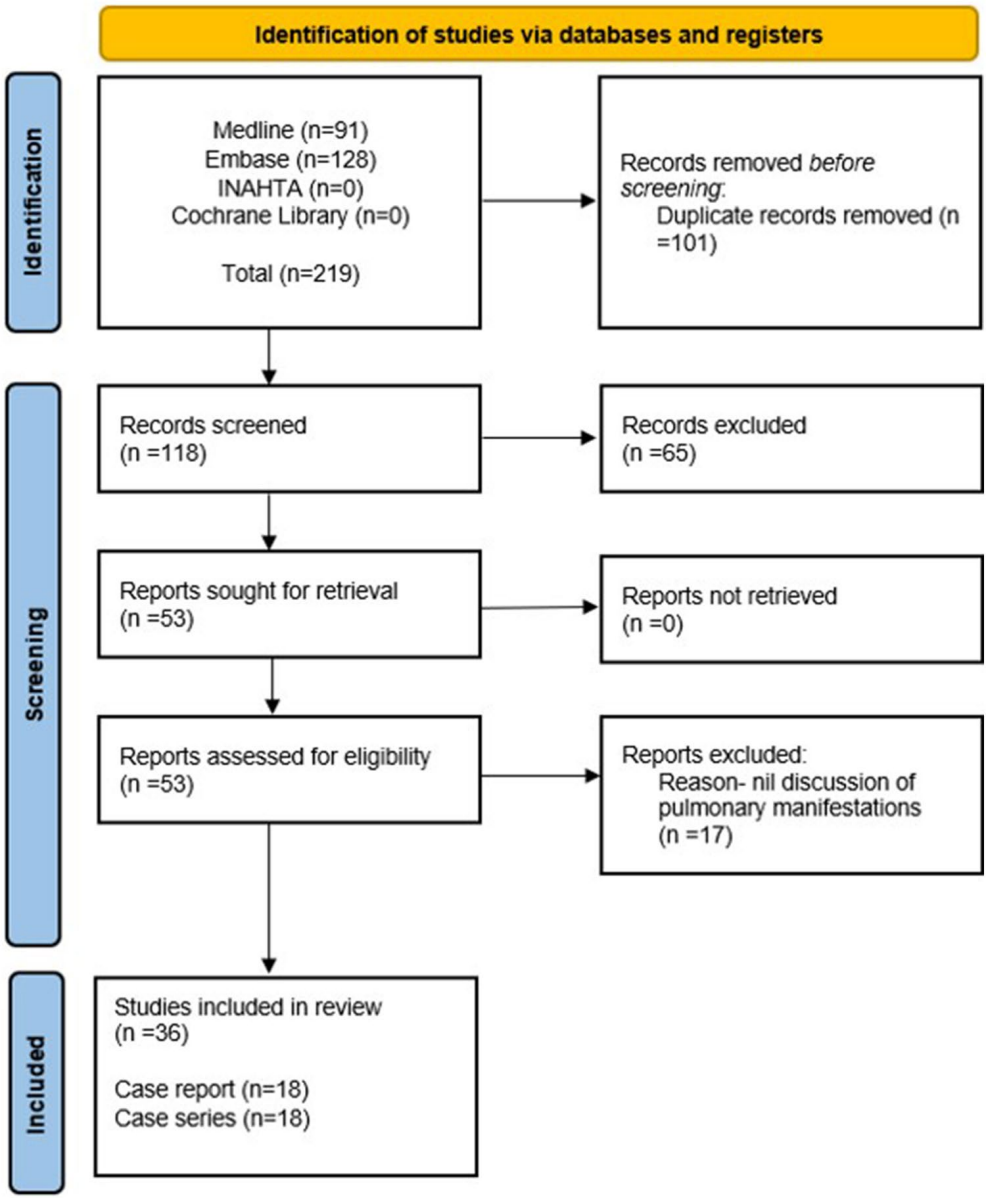
Flow diagram of stages of systematic literature review. *INAHTA* International Network of Agencies for Health Technology Assessment. Cochrane Library encompasses library of: systematic reviews; systematic review protocols; controlled clinical trials

Initially, 219 articles were retrieved with 36 ultimately included (18 case reports, 18 case series; Fig. 1). A pooled total of 269 patients with VEXAS were included, 98.6% of whom were male, with an overall mean age of 66.8 years (SD 7.3) at disease onset. Cases were from: Europe (*n* = 181); North America (62); South America (*n* = 1); Asia (*n* = 21); Australasia (*n* = 4).

Demographics and extracted clinical information are summarized in Table 1. 56.1% of included patients had pulmonary involvement at presentation. The most frequently described manifestation was pulmonary infiltrates (43.1%; *n* = 116) [1, 8, 12–34], followed by pleural effusion (7.4%; *n* = 20) [8, 18, 20, 22, 24, 27, 28, 32, 35] and idiopathic interstitial pneumonia (3.3%; *n* = 9) [14, 18, 25, 27, 28, 32, 36, 37]. Other pulmonary manifestations described were NSIP (*n* = 1) [14]; bronchiolitis obliterans (*n* = 3) [14]; pulmonary vasculitis (*n* = 6) [14, 24]; bronchiectasis (*n* = 1) [14]; alveolar haemorrhage (*n* = 1) [38]; pulmonary embolism (*n* = 4) [35, 39–41]; bronchial stenosis (*n* = 1) [42]; and alveolitis (*n* = 1) [36].

**Table 1:** Article information, cohort demographics, and pulmonary manifestations, including sample size (*n*) for each type

Article information			Sample size and demographics			Overall pulmonary involvement		Pulmonary manifestations										
Source	Country of study population	Type of study	Sample size (with VEXAS)	Male (%)	Mean age at onset (years)	Pulmonary involvement (*n*)	Pulmonary involvement (%)	Infiltrates (*n*)	Idiopathic interstitial pneumonia (*n*)	NSIP (*n*)	Bronchiolitis obliterans (*n*)	Pulmonary vasculitis (*n*)	Bronchiectasis (*n*)	Pleural effusion (*n*)	Alveolar haemorrhage (*n*)	PE (*n*)	Bronchial stenosis and wall thickening (*n*)	Alveolitis (*n*)
Van der Made 2022 [14]	Netherlands	Case series	12	100	67	8	66.7	1	1	1	3	5	1	0	0	0	0	0
Shaukat 2022 [20]	USA	Case report	1	100	80	1	100	1	0	0	0	0	0	1	0	0	0	0
Sharma 2022 [21]	India	Case report	1	100	70	1	100	1	0	0	0	0	0	0	0	0	0	0
Pamies 2022 [23]	Spain	Case report	1	100	76	1	100	1	0	0	0	0	0	0	0	0	0	0
Muratore 2022 [32]	Italy	Case series	3	100	67.7	3	100	2	0	0	0	1	0	1	0	0	0	0
Matsumoto 2022 [38]	Japan	Case report	1	100	60	1	100	1	0	0	0	0	0	0	1	0	0	0
Matsubara 2022 [25]	Japan	Case report	1	100	68	1	100	1	1	0	0	0	0	0	0	0	0	0
Martin-Nares 2022 [26]	Mexico	Case report	1	100	77	1	100	1	0	0	0	0	0	0	0	0	0	0
Loschi 2022 [39]	France	Case report	1	100	60	1	100	0	0	0	0	0	0	0	0	1	0	0
Li 2022 [27]	USA	Case series	2	100	65	1	50	1	1	0	0	0	0	1	0	0	0	0
Kao 2022 [22]	USA	Case report	1	100	50	1	100	1	0	0	0	0	0	1	0	0	0	0
Islam 2022 [28]	Australia	Case series	3	100	67.7	2	66.7	2	2	0	0	0	0	2	0	0	0	0
Goyal 2022 [29]	USA	Case report	1	100	64	1	100	1	0	0	0	0	0	0	0	0	0	0
Georgin-Lavialle 2022 [8]	France	Case series	116	95.7	71	57	49.1	47	0	0	0	0	0	11	0	0	0	0
Diarra 2022 [30]	France	Case series	6	100	52.7	2	33.3	2	0	0	0	0	0	0	0	0	0	0
Ciferska 2022 [31]	Czech Republic	Case series	3	100	72.7	1	33.3	1	0	0	0	0	0	0	0	0	0	0
Beaumesnil 2022 [42]	France	Case series	3	100		1	33.3	0	0	0	0	0	0	0	0	0	1	0
Afsahi 2022 [32]	USA	Case series	2	100	75.5	1	50	1	1	0	0	0	0	1	0	0	0	0
Zakine 2021 [44]	France	Case series	8	100		2	25	0	0	0	0	0	0	0	0	0	0	0
Tsuchida 2021 [43]	Japan	Case series	14	85.7	72.1	7	50	0	0	0	0	0	0	0	0	0	0	0
Thomas 2021 [33]	USA	Case report	1	100	62	1	100	1	0	0	0	0	0	0	0	0	0	0
Takahashi 2021 [34]	Japan	Case report	1	100	55	1	100	1	0	0	0	0	0	0	0	0	0	0
Staels 2021 [7]	Belgium	Case series	2	100	72.5	0	0	0	0	0	0	0	0	0	0	0	0	0
Sakuma 2021 [12]	Japan	Case report	1	100	61	1	100	1	0	0	0	0	0	0	0	0	0	0
Poulter 2021 [13]	UK	Case series	7	85.7	72.7	2	28.5	2	0	0	0	0	0	0	0	0	0	0
Poulter 2021 [15]	UK	Case series	10	100	70	2	20	2	0	0	0	0	0	0	0	0	0	0
Oo 2021 [40]	Singapore	Case report	1	100	69	1	100	0	0	0	0	0	0	0	0	1	0	0
Obiorah 2021 [16]	USA	Case series	16	100	60.6	14	87	14	0	0	0	0	0	0	0	0	0	0
Lotscher 2021 [17]	Switzerland	Case report	1	100	68	1	100	1	0	0	0	0	0	0	0	0	0	0
Lee 2021 [41]	Singapore	Case report	1	100	69	1	100	0	0	0	0	0	0	0	0	1	0	0
Himmelmann 2021 [18]	Switzerland	Case report	1	100	77	1	100	1	1	0	0	0	0	1	0	0	0	0
Grey 2021 [35]	Australia	Case report	1	100	74	1	100	0	0	0	0	0	0	1	0	1	0	0
Ferrada 2021 [19]	USA and UK	Case series	13	100	59.8	10	77	10	0	0	0	0	0	0	0	0	0	0
Delplanque 2021 [36]	France	Case series	6	83	66.1	2	33.3	0	1	0	0	0	0	0	0	0	0	1
Dehghan 2021 [37]	UK	Case report	1	100	55	1	100	0	1	0	0	0	0	0	0	0	0	0
Beck 2020 [1]	USA and UK	Case series	25	100	62.6	18	72	18	0	0	0	0	0	0	0	0	0	0

*PE* pulmonary embolism

With regards to co-existing autoimmune and/or autoinflammatory diseases, pulmonary involvement was described in patients previously diagnosed with or meeting diagnostic criteria for several conditions: relapsing polychondritis (*n* = 33) [14, 19, 23, 31, 41–43]; ANCA-associated-vasculitis (AAV; *n* = 1) [24]; antibody-negative vasculitis (*n* = 9) [14]; IgA-vasculitis (*n* = 1) [23]; systemic lupus erythematosus (*n* = 1) [21]; Sweet syndrome (*n* = 2) [25, 44]; Behcet’s disease (*n* = 1) [38]. Pulmonary involvement in patients with these co-existing conditions, where described, are summarized in Table 1.

## Discussion

Pulmonary manifestations are commonly described in patients with VEXAS syndrome, with over 50% of those included in our review having at least one type of lung pathology at initial presentation. Alongside skin lesions and haematologic manifestations, these are the most frequently described features of VEXAS and a leading cause of mortality in these patients [1, 2].

### Pulmonary manifestations

The most commonly described manifestation was pulmonary infiltrates, present in 43% of cases included in our review, often co-existing with other lung pathologies such as NSIP, pulmonary vasculitis and pleural effusion [14]. A cohort study of 116 patients from the French multicentre registry, one of the largest case series in our review, also noted lung pathologies to be one of the most common clinical features of VEXAS and associated with mortality, with pulmonary infiltrates the most frequently described manifestation [8]. When assessing phenotype–genotype correlations, lung infiltrates were more common in those with the UBA1 p.Met41Thr or p.Met41Val mutations, compared with p.Met41Leu [8]. A separate study also found these two mutations to have increased association with pulmonary involvement [24].

Pulmonary embolism was identified in four cases included in our review, one co-existing with haemophagocytic lymphohistiocytosis, another with Kikuchi-Fujimoto disease (an autoinflammatory condition characterized by necrotising lymphadenitis and fever) [35, 41]. Incidence of venous thromboembolic events in VEXAS has been reported as being as high as 36% while arterial thrombotic events have an estimated incidence of 1.6% [40]. This is in part likely due to decreased ubiquitylation as a result of UBA1 gene mutation, leading to chronic inflammation and haemostatic and endothelial dysfunction [40]. As with immunosuppression, prophylaxis and treatment of thrombotic events in patients with VEXAS requires further study.

### Previous and coexisting autoimmune conditions

Our review identified several co-existing autoimmune and/or autoinflammatory conditions, with predominance of respiratory involvement, in patients diagnosed with VEXAS syndrome, most commonly relapsing polychondritis (*n* = 33). Patients had typical pulmonary manifestations seen in this condition, such as bronchial stenosis and bronchial wall thickening [42]. VEXAS was also diagnosed in patients with vasculitis (including Behcet’s syndrome), in some cases in conjunction with relapsing polychondritis [23]. VEXAS syndrome shares many features of vasculitis, not only pulmonary involvement, but also skin, haematological and ocular features, as well as preponderance in people of an older age. We identified one case of VEXAS in ANCA-associated vasculitis, which was characterized by pulmonary vasculitis in addition to rapidly progressive renal failure with necrotising glomerulonephritis, inflammatory manifestations and myelodysplastic features, resistant to multiple forms of immunosuppression (including cyclophosphamide and rituximab) and requiring high-dose corticosteroids to induce remission [24]. In the case of Behcet’s syndrome co-existing with VEXAS, myelodysplastic features were also present, with ground-glass changes seen on computer tomography (CT) scanning, characteristic of several cases included in our review [38]. This is not unusual in patients with Behcet’s, but it raises the need to consider VEXAS and UBA1 genetic variants especially in males with myelodysplasia.

### Points to consider in treatment

Early identification of VEXAS in patients with other autoinflammatory conditions not only aids diagnosis but, importantly, can expedite management of the condition. Treatment of VEXAS is an evolving area of research and it is beyond the scope of this article to be able to comment on potential therapeutic options from the evidence presented here. Treatment strategies used in the included cases varied markedly, and included high-dose corticosteroids (most commonly reported), conventional synthetic disease-modifying antirheumatic drugs (DMARDs) including methotrexate and mycophenolate, and biologic DMARDS, including cyclophosphamide, IL-1 antagonists, IL-6 inhibitors, TNF inhibitors, rituximab and JAK inhibitors [1, 8, 24, 26, 29, 32, 35, 42]. There was one reported case of the use of ruxolitinib followed by allogeneic stem cell transplant [39]. It was often unclear in many of the cases, especially in series, as to which patients received which therapy, or were reported to have cycled through multiple agents. If reported, it was unclear whether the pulmonary manifestations were a factor in deciding which treatment to administer. Certainly, the use of methotrexate, for example, suggests that pulmonary disease was less of a concern in these particular patients. The heterogeneity of reported therapies demonstrates the many possible disease presentations and organ involvement, as well as the fact that this remains an active area of research. Similar to many autoinflammatory conditions, however, high-dose corticosteroids were the most commonly administered and effective treatment, as reported in the first article by Beck et al. [1]. Therapeutic strategies from conditions related to VEXAS may aid its management especially where there is pulmonary involvement given this is a leading cause of morbidity and mortality in these patients.

## Conclusion

In conclusion, pulmonary disease is common in patients with VEXAS, most frequently described as pulmonary infiltrates. To our knowledge, this is the first systematic review conducted in patients with VEXAS syndrome, including on one of its commonest manifestations, lung involvement. It is unclear if respiratory manifestations are part of the primary disease or a separate pathology (including those predating the development of VEXAS). Based on our review it is also not clear whether those with pulmonary manifestation had any history of chronic lung conditions or not, which, if present, will likely skew respiratory outcomes and morbidity.

Our data will aid consideration of VEXAS in those with other autoimmune and autoinflammatory conditions reported in the literature, and can help to guide management strategies in this diverse patient cohort. Further epidemiological and larger cohort analyses in VEXAS patients are required to aid further characterisation of pulmonary involvement and disease management.

## Supplementary Information

Below is the link to the electronic supplementary material.

Supplementary file1 (DOCX 12 KB)

## Data Availability

Data available upon request.

## References

[1] Beck DB, Ferrada MA, Sikora KA, Ombrello AK, Collins JC, Pei W (2020). Somatic mutations in UBA1 and severe adult-onset autoinflammatory disease. N Engl J Med.

[2] Grayson PC, Patel BA, Young NS (2021). VEXAS syndrome. Blood.

[3] Jamshidi A, Aslani S, Mahmoudi M (2018) Pulmonary manifestations of autoinflammatory disorders. Pulm Manif Prim Immunodefic Dis 193–211

[4] Gulati M, Mani NBS, Singh P, Suri S (2001). Pulmonary manifestations of Behçet’s disease. Thorax.

[5] Dubey S, Gelder C, Pink G, Ali A, Taylor C, Shakespeare J, et al. (2021) Respiratory subtype of relapsing polychondritis frequently presents as difficult asthma: a descriptive study of respiratory involvement in relapsing polychondritis with 13 patients from a single UK centre. ERJ Open Res 7(1):00170–2020. 10.1183/23120541.00170-2020 10.1183/23120541.00170-2020 PMC7882783 33614776

[6] Casal Moura M, Baqir M, Tandon Y, Samec M, Reichard K, Mangaonkar A (2022). POS1377 LUNG INVOLVEMENT IN VEXAS SYNDROME. Ann Rheum Dis.

[7] Staels F, Betrains A, Woei-A-Jin FJSH, Boeckx N, Beckers M, Bervoets A (2021). Case report: VEXAS syndrome: from mild symptoms to life-threatening macrophage activation syndrome. Front Immunol.

[8] Georgin-Lavialle S, Terrier B, Guedon AF, Heiblig M, Comont T, Lazaro E (2022). Further characterization of clinical and laboratory features in VEXAS syndrome: large-scale analysis of a multicentre case series of 116 French patients*. Br J Dermatol.

[9] (2022) A review of VEXAS syndrome in 116 French patients. Br J Dermatol 186: e115–e115. 10.1111/bjd.20989

[10] Cochrane Handbook for Systematic Reviews of Interventions | Cochrane Training. https://training.cochrane.org/handbook/current. Accessed 31 Aug 2020

[11] Page MJ, Moher D, Bossuyt PM, Boutron I, Hoffmann TC, Mulrow CD, et al. (2021) PRISMA 2020 explanation and elaboration: updated guidance and exemplars for reporting systematic reviews. BMJ. 372. https://www.bmj.com/content/372/bmj.n160. Accessed 3 Oct 2021 10.1136/bmj.n160 PMC8005925 33781993

[12] Sakuma M, Tanimura A, Yasui S, Ishiguro K, Kobayashi T, Ohshiro Y (2021). A Case of polychondritis-onset refractory organizing pneumonia with cytopaenia diagnosed as VEXAS syndrome: the disease course of 7 years. Rheumatology.

[13] Poulter J, Gough A, Isaacs JD, Green M, McHugh N, Hordon L (2022). A high-throughput amplicon screen for somatic UBA1 variants in cytopenic and giant cell arteritis cohorts. J Clin Immunol.

[14] van der Made CI, Potjewijd J, Hoogstins A, Willems HPJ, Kwakernaak AJ, de Sevaux RGL (2022). Adult-onset autoinflammation caused by somatic mutations in UBA1: a Dutch case series of patients with VEXAS. J Allergy Clin Immunol.

[15] Poulter JA, Collins JC, Cargo C, De Tute RM, Evans P, Ospina Cardona D (2021). Novel somatic mutations in UBA1 as a cause of VEXAS syndrome. Blood.

[16] Obiorah IE, Patel BA, Groarke EM, Wang W, Trick M, Ombrello AK (2021). Benign and malignant hematologic manifestations in patients with VEXAS syndrome due to somatic mutations in UBA1. Blood Adv.

[17] Lötscher F, Seitz L, Simeunovic H, Sarbu AC, Porret NA, Feldmeyer L (2022). Case report: genetic double strike: VEXAS and TET2-positive myelodysplastic syndrome in a patient with long-standing refractory autoinflammatory disease. Front.

[18] Himmelmann A, Brücker R (2021). The VEXAS syndrome: uncontrolled inflammation and macrocytic anaemia in a 77-year-old male patient. Eur J Case Rep Intern Med.

[19] Ferrada MA, Sikora KA, Luo Y, Wells KV, Patel B, Groarke EM (2021). Somatic mutations in UBA1 define a distinct subset of relapsing polychondritis patients with VEXAS. Arthritis Rheumatol.

[20] Shaukat F, Hart M, Burns T, Bansal P (2022). UBA1 and DNMT3A mutations in VEXAS syndrome. A case report and literature review. Mod Rheumatol Case Reports..

[21] Sharma A, Naidu G, Deo P, Beck DB (2022). VEXAS syndrome with systemic lupus erythematosus: expanding the spectrum of associated conditions. Arthritis Rheumatol.

[22] Kao RL, Jacobsen AA, Billington CJ, Yohe SL, Beckman AK, Vercellotti GM (2022). A case of VEXAS syndrome associated with EBV-associated hemophagocytic lymphohistiocytosis. Blood Cells, Mol Dis.

[23] Pàmies A, Ferràs P, Bellaubí-Pallare N, Gimenez T, Raventós A, Colobran R (2022). VEXAS syndrome: relapsing polychondritis and myelodysplastic syndrome with associated immunoglobulin A vasculitis. Rheumatol (United Kingdom).

[24] Muratore F, Marvisi C, Castrignanò P, Nicoli D, Farnetti E, Bonanno O (2022). VEXAS syndrome: a case series from a single-center cohort of italian patients with vasculitis. Arthritis Rheumatol.

[25] Matsubara A, Tsuchida N, Sakurai M, Maeda A, Uchiyama Y, Sasaki K (2022). A case of VEXAS syndrome with sweet’s disease and pulmonary involvement. J Dermatol.

[26] Martín-Nares E, Vargas-Serafín C, Delgado-de la Mora J, Montante-Montes de Oca D, Grayson PC, Larios E (2022). Orbital and periorbital inflammation in VEXAS syndrome. Scand J Rheumatol.

[27] Li P, Venkatachalam S, Cordona DO, Wilson L, Kovacsovics T, Moser KA (2022). A clinical, histopathological, and molecular study of two cases of VEXAS syndrome without a definitive myeloid neoplasm. Blood Adv.

[28] Islam S, Cullen T, Sumpton D, Damodaran A, Heath D, Bosco A (2022). VEXAS syndrome: lessons learnt from an early Australian case series. Intern Med J.

[29] Goyal A, Narayanan D, Wong W, Laga AC, Connell NT, Ritter SY (2022). Tocilizumab for treatment of cutaneous and systemic manifestations of vacuoles, E1 enzyme, X-linked, autoinflammatory, somatic (VEXAS) syndrome without myelodysplastic syndrome. JAAD Case Reports.

[30] Diarra A, Duployez N, Fournier E, Preudhomme C, Coiteux V, Magro L (2022). Successful allogeneic hematopoietic stem cell transplantation in patients with VEXAS syndrome: a 2-center experience. Blood Adv.

[31] Ciferská H, Gregová M, Klein M, Šenolt L, Maaloufová JS, Pavelka K (2022). VEXAS syndrome: a report of three cases. Clin Exp Rheumatol.

[32] Afsahi V, Christensen RE, Alam M. (2022) VEXAS syndrome in dermatology. Arch Dermatol Res. https://pubmed.ncbi.nlm.nih.gov/35201420/. Accessed 26 Sep 2022 10.1007/s00403-022-02340-4 35201420

[33] Thomas VT, Penmetcha M (2021). Myelodysplastic syndrome associated with auto-immune inflammatory disease in VEXAS syndrome. J Hematol.

[34] Takahashi N, Takeichi T, Nishida T, Sato J, Takahashi Y, Yamamura M (2021). Extensive multiple organ involvement in VEXAS syndrome. Arthritis Rheumatol (Hoboken, NJ).

[35] Grey A, Cheong PL, Lee FJ, Abadir E, Favaloro J, Yang S (2021). A case of VEXAS syndrome complicated by hemophagocytic lymphohistiocytosis. J Clin Immunol.

[36] Delplanque M, Aouba A, Hirsch P, Fenaux P, Graveleau J, Malard F (2021). USAID associated with myeloid neoplasm and VEXAS syndrome: two differential diagnoses of suspected adult onset still’s disease in elderly patients. J Clin Med.

[37] Dehghan N, Marcon KM, Sedlic T, Beck DB, Dutz JP, Chen LYC (2021). Vacuoles, E1 enzyme, X-linked, autoinflammatory, somatic (VEXAS) syndrome: fevers, myalgia, arthralgia, auricular chondritis, and erythema nodosum. Lancet.

[38] Matsumoto H, Asano T, Tsuchida N, Maeda A, Yoshida S, Yokose K (2022). Behçet’s disease with a somatic UBA1 variant: Expanding spectrum of autoinflammatory phenotypes of VEXAS syndrome. Clin Immunol.

[39] Loschi M, Roux C, Sudaka I, Ferrero-Vacher C, Marceau-Renaut A, Duployez N (2022). Allogeneic stem cell transplantation as a curative therapeutic approach for VEXAS syndrome: a case report. Bone Marrow Transplant.

[40] Oo TM, Koay JTJ, Lee SF, Lee SMS, Lim XR, Fan BE (2022). Thrombosis in VEXAS syndrome. J Thromb Thrombolysis.

[41] Lee SMS, Fan BE, Lim JHL, Goh LL, Lee JSS, Koh LW (2021). A case of VEXAS syndrome manifesting as Kikuchi-Fujimoto disease, relapsing polychondritis, venous thromboembolism and macrocytic anaemia. Rheumatology (Oxford).

[42] Beaumesnil S, Boucher S, Lavigne C, Urbanski G, Lacombe V (2022). Ear, nose, throat, and bronchial involvements in VEXAS syndrome: specifying the spectrum of clinical features. JAMA Otolaryngol Neck Surg.

[43] Tsuchida N, Kunishita Y, Uchiyama Y, Kirino Y, Enaka M, Yamaguchi Y (2021). Pathogenic UBA1 variants associated with VEXAS syndrome in Japanese patients with relapsing polychondritis. Ann Rheum Dis.

[44] Zakine E, Schell B, Battistella M, Vignon-Pennamen MD, Chasset F, Mahévas T (2021). UBA1 variations in neutrophilic dermatosis skin lesions of patients with VEXAS syndrome. JAMA Dermatol.

