# Supplementary material for: Pulmonary manifestations in VEXAS (vacuoles, E1 enzyme, X-linked, autoinflammatory, somatic) syndrome: a systematic review
Source: Rheumatol Int. 2023 Jan 8;43(6):1023–32. doi: 10.1007/s00296-022-05266-2 (PMC10126013; doi:10.1007/s00296-022-05266-2)
Supplement: Supplementary file 1 — Supplementary file1 (DOCX 12 KB) [file 296_2022_5266_MOESM1_ESM.docx]

**Search strategies**

**A.1 Medline**

Ovid MEDLINE® ALL <1946 to May 11, 2022> Results per line

Date: 13/05/2022

1 vexas.mp. 91

2 “Vacuoles, E1 enzyme, X-linked, Autoinflammatory, Somatic”.mp. 33

3 1 or 2 91

**A.2 Embase**

Embase <1974 to 2022 May 12> Results per line

Date: 13/05/2022

1 vexas.mp. 127

2 “Vacuoles, E1 enzyme, X-linked, Autoinflammatory, Somatic”.mp. 52

3 1 or 2 128

**A.3 Cochrane**

Cochrane Central Register of Controlled Trials (CENTRAL) and Cochrane Database of Systematic Reviews (CDSR)

Results per line

Date: 13/05/2022

#1 (vexas):ti,ab,kw

0

#2 (Vacuoles NEXT E1 enzyme NEXT X-linked NEXT Autoinflammatory

NEXT Somatic):ti,ab,kw

0

#3 #1 OR #2

0
